# Human Papillomavirus Condylomas of Extragenital Site in Immunocompetent Man, New York, USA, 2025

**DOI:** 10.3201/eid3210.260872

**Published:** 2026-10

**Authors:** Sabrina Fong, John G. Zampella

**Affiliations:** New York University Langone Health, Preston Robert Tisch Center for Men’s Health, New York, New York, USA

**Keywords:** human papillomavirus 6, human papillomavirus 11, condylomas, condylomata acuminata, papillomavirus infections, cutaneous, viruses, New York City, New York, United States

## Abstract

Human papillomavirus types 6 and 11 are associated with mucosal condylomata acuminata, with rare reports of extragenital manifestations. We report a case of condyloma localized to the neck of an immunocompetent heterosexual man, without concurrent anogenital lesions. Our findings challenge theories of the site-specific tropism of these human papillomavirus types.

Human papillomavirus (HPV) types 6 and 11 have long been associated with condylomata acuminata, accounting for 90% of warts affecting the anogenital region (*1*). History of immunosuppression, both primary or secondary, is associated with HPV-related diseases, making affected patients susceptible to development of cutaneous and mucosal warts (*2*). Reports of extragenital manifestations of condylomas are rare, with previous cases being associated with pediatric populations, men who have sex with men, and persons with a history of HIV. We report a case of HPV types 6 and 11–positive condylomas localized to the lateral neck of an immunocompetent heterosexual man without concurrent anogenital involvement.

In 2025, a heterosexual man in his 40s with an unremarkable medical history sought treatment for a growth on the right side of his neck present for 1 year prior, with reported increase in size. On examination, we noted 6 clustered verrucous-to-mamillated brown papules distributed along the right lateral neck (Figure). Our initial differential diagnosis included inflamed seborrheic keratoses and verruca vulgaris. We treated the lesions with cryotherapy, which provided temporary resolution. The patient returned to our clinic 11 months later for recurrence. We performed a shave biopsy, which showed papillated epidermal hyperplasia with hypergranulosis, compact orthokeratosis, and dilated blood vessels in the papillary dermis. HPV testing by in situ hybridization was positive for HPV types 6 and 11. Screening tests for sexually transmitted infections revealed negative results for gonorrhea, chlamydia, syphilis, and HIV. Complete blood count testing and a comprehensive metabolic panel also yielded results that were within reference limits. Immunologic screening revealed a positive antinuclear antibody titer of 1:80 (reference <1:40) but unremarkable levels of IgA, IgG, and IgM.

**Figure:**
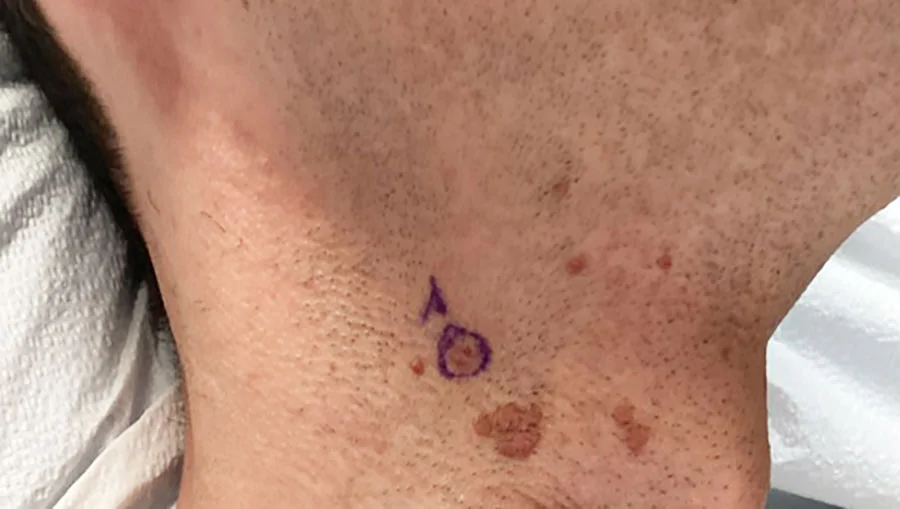
Image from a case report of condylomas caused by human papillomavirus on extragenital cutaneous site in immunocompetent man, New York, USA, 2025. Brown, clustered, verrucous-to-mamillated papules are shown on the right lateral neck. The circled lesion underwent biopsy.

Our case report challenges the traditional paradigm of HPV types 6 and 11 tropism to mucosal skin (*3*). Although reports do exist describing condylomas at extragenital cutaneous sites, those reports have largely involved children, patients with immunosuppression, or persons with concurrent genital involvement (*4*–*6*). One report described a patient with extragenital cutaneous HPV type 6 without genital involvement; however, the patient had a history of HIV (*6*). Although reports of extragenital condylomas and cutaneous HPV types 6 infection exist, cases of HPV types 6 and 11–positive extragenital cutaneous lesions in immunocompetent adults without concurrent anogenital disease are rare.

HPV is known to affect mucosal tissue, particularly of the head and neck, with HPV types 16 accounting for most oropharyngeal squamous cell carcinomas (*7*). Of interest, HPV types 6 and 11 can be co-transmitted with higher-risk types of HPV in genital skin (*8*,*9*). Hypotheses for transmission to this area of the neck include autoinoculation, fomites, skin-to-skin contact with mucosal skin, or even vertical transmission (*10*). A limitation in our case report was the inability to distinguish between HPV types 6 and 11 based on laboratory in situ hybridization technique. Nonetheless, this case highlights the possibility of condylomas in nonanogenital cutaneous sites in immunocompetent hosts, emphasizing that practitioners should consider biopsy with HPV typing in atypical verrucous lesions on cutaneous sites.
